# History of the Linear Extraction of Cataract*Translated from the German by the author, with the kind assistance of Albert Scheffer, Esq., St. Paul.

**Published:** 1872-11

**Authors:** B. B. Schwarzbach

**Affiliations:** St. Paul, Minn.


Article III.—*History of the Linear Extraction of Cataract.** By B. B. Schwarzbach, M.D., St. Paul, Minn.

It cannot be denied that no branch of practical medicine has made such enormous advances during the last few years as ophthalmology. The ophthalmoscope has lifted the veil which hitherto, in most cases, had proved a bar to the diagnosis of pathological processes in the interior of the eye. In surgical therapy, the linear extraction of cataract is a most important and beneficent acquisition.

It may be interesting to give in a few words the history of the modified linear extraction, as it is now almost universally acknowledged and practiced. The first reliable statements on record in regard to the extraction of cataract by a linear cut are from the last century. Joseph Palucci recommends a linear incision in the cornea in order to remove membranous opacities and contracted lenses. F. von Jaeger, who recognized that the linear cut is by far less dangerous (even in cases where the production of matter followed the operation) than the flap cut, (which is always precarious), adopted this method. After him, Gibson, of Manchester, proposed the adoption of the linear cut as the chief method in the operation for all soft cataracts. Soon after he dropped the idea, because the "spooning" of the substance of the lens from out the pupil is seldom sufficient to remove the more solid centre, and he practiced the linear extraction only as an after-operation of discisio cataractæ. This entire method seeming unfit to remove the whole lens system was soon forgotten, until von Graefe re-adopted the almost banished

* Translated from the German by the author, with the kind assistance of Albert Scheffer, Esq., St. Paul.

linear cut. Even he only made use of the same after discisio cataractæ. Having, however, become convinced of the success of a linear cut of 2½‴, necessitated by the swelling of the lens after discisio, he declared the same adapted to remove certain cataracts in the shortest and safest manner. As contra-indication, von Graefe pronounced the normal consistency of the lens (because the tough substance of the centre prevents evacuation) and unripeness and hardness of the cataract, as unqualified indications of the softness of the lens. As yet no important step had been made in the more general adoption of the linear extraction, because the operation is necessarily followed by the direct irritation of the iris, and especially by such as is caused by the remaining of the lens. Von Graefe attempted the removal of that portion of the iris which is unavoidably affected, in view of the acknowledged anti-phlogistical result of the excision of an iris portion in glaucoma and irido-choroiditis. The benefits of this were:

1st. The prevention of the inflammatory infiltration.

2nd. Of the so often annoying "prolapsus iridis."

3rd. The possibility of bringing the spoon behind the centre of the lens without pressing its body aside.

This procedure was not, as already stated, applicable in all forms of cataract. Undoubtedly in cases where the inner cornear cut extends only 3‴, while the larger centres of lenses reach the proportion of 3¾‴ and more, it is evident that the process of extraction causes more or less serious bruises to the parts cut. Such cases always require the flap extraction.

Encouraged by the experiments of Critchet and Bowman in London, who in cases of the extraction of the largest and hardest cataracts, resorted to a cut extending over the third part of the periphery of the cornea, von Graefe combined the larger cut with the more beneficial healing. The cut in the periphery of the cornea, as practiced by Critchet and Bowman, though sufficiently large (3¾‴), did not dispense with the disadvantage in the healing of the common flap cut. It was therefore necessary, while retaining the linear cut, to apply the same as nearly as possible to the periphery of the cornea in the sclera. In order to attain this end it became imperative to dispense with the use of the lancet, as had been customary, and replace the same by a punction and contra-punction cut, with a knife hardly the width of 1‴, ending with a perpendicular cut to the surface of the cornea, thus effecting an almost exact linear opening. Compared with a flap created by a cut of the lancet of equal length, the application of this method produced one of comparatively small dimensions. The periphericity of the wound brings forth only a slight reaction, which is still lessened by the formation of a flap of conjunctiva, thus securing to the healing of the sclera-cornea wound all benefits of the sub-conjunctiva process. The difference between the channels of the wound produced by this cut, as compared with the one effected by the lancet, lies chiefly in the fact that the first mentioned attains an almost perpendicular direction to the surface of the cornea, in contra-distinction to the cut of the lancet. This perpendicular direction undoubtedly secures more beneficial terms for the evacuation of the lens than the other.

This makes it possible to remove the lens without instruments, or at least with such as cause less damage than those formerly in use. The realization of this possibility was now made the first requirement of the linear extraction. If, as was hitherto the case, in the disadvantageous condition of the inner wound to the evacuation of the lens, it became necessary to make use of large and reliable instruments, it was now sufficient to use smaller ones and those less damaging to the surroundings, for the same purpose and with better effect. Therefore von Graefe replaced the spoon by the hook, the application of which in the rear of the corticalis produced no detrimental pressure. On the other hand, the infancy of the process warranted the expectation of an improvement in the technicality, thus securing the probability of lessening the mishaps. The disadvantage of linear extraction in forming an artificial pupil cannot be denied; but von Graefe, by placing the coloboma of the iris, in spite of the increased difficulty, to the upper part of the eye, gained a decided advantage, both in a cosmetic sense, and an improved function of the organ. Thus, the iridectomy was not performed prior to the extraction, as was the case in the flap cut, but *uno tempore* with the same.

This modified linear extraction now became the object of further investigation and experiments, because its adaptability to the universal method defied contradiction. Especially did von Graefe practice numerous extractions in this manner, and though often operating under very discouraging circumstances, called forth by the heat of summer, and the condition of the hospital and nursing departments, the results were highly satisfactory. With the exception of cataract congenita, partial cataract, and reacting cataract formation, in which cases discisio proved a more simple and commendable process, all other forms were removed by the modified linear extraction. Out of 300 cases, 94 per cent. were a complete success.

The improvement in the technicality, and the introduction of the sliding process, materially lessened the so-much-dreaded "prolapsus corpus vitrei," so that in comparing the results of this method with the flap extraction, the former proved the more preferable. The shortening of the after treatment, and the less scrupulous nursing of the patient after operation, was a further advantage in linear extraction. The application of chloroform was resorted to in exceptional cases only; a complete narcosis causes too much loss of time, and hinders the action of the muscles of the patient, which is desirable in cases of adherent corticalis, especially in the after cleansing of the pupil. Chloroform was administered only in cases of abnormal stiffness of the eyelids, prominence of the eye, increased reflectory irritation, and timidity of the patient.

The numerous operations superintended by von Graefe proved in favor of the modified linear extraction, by some important rules influencing the technical process. Regarding the flap of the conjunctiva, which greatly accelerates the healing of the wound, it was found that in general the difference in height was of little importance; still, very large flaps, exceeding the dimensions of $1\frac{1}{2}'''$, are not favorable to the healing, and therefore not recommendable, and extension of the flap from the centre is the most practicable. A further caution in the operation is required by the iridectomy. If, in the excision of the iris, the most scrupulous care is not exercised, a growing fast of the corners of same in the channel of the wound, with the necessary consequences, may be looked for. A radical excision of the iris sphincter in the boundary of the external wound is therefore necessary. Experience further showed that the "sliding movement" in the evacuation of the lens produced a far smaller per centage (4 per cent.) of prolapsus corpus vitrei than the application of the hook (10 per cent.) In the first mentioned method a much more complete removal of the corticalis is effected than in the use of any traction instruments; exceptions are extremely hard cataracts, the evacuation of which is much simplified by the traction method, thereby lessening the pressure. Thus the linear extraction gained more and more supporters, because the results proved that it was not only destined to replace the flap extraction in isolated cases, but to entirely supersede it. All renowned oculists unanimously promulgated the fact, that with the modified linear extraction, a new era in the practice of cataract operation had come.

But, however great the progress attained by the linear extraction through the modifications achieved, numerous as were its supporters in its present form, the disadvantages produced by the traction instruments of whatever shape, soon called forth the wish to discover a method which would dispense with every such instrument. Experiments to this end made by von Graefe, proved that the evacuation of the lens was possible by a pressure applied from without, after splitting the capsula of the lens by the cystotome. Of course the sliding process, which in one case out of every eight of cataract always required the application of a traction instrument, was not sufficient to attain this end, because the larger part of the force applied acts principally as an acceleration of the inter-ocular pressure, without aiding the evacuation of the cataract in preparing its course. This mainly opposed the removal of compact lenses closely adhering to the capsula, because in these cases the sliding movement could only effect a slow and difficult evacuation. The lens must necessarily enter the channel of the wound by the corresponding position of the inner wound and the outline of the lens, provided the pressure is properly applied. This somewhat tampons the wound, and lessens the possibility of prolapsus corpus vitrei.

Von Graefe proved the value of this improvement as introduced by him in his publication of same, through the results achieved from two hundred and thirty operations, and concludes that:

1st. All forms of cataract can be extracted with great precision, avoiding a contusion of the wound, as it still occurred in the sliding movement.

2nd. Owing to the equal filling in of the wound by the lens, the formation of prolapsus was of less frequent occurrence than formerly.

3rd. In most cases a complete evacuation of the corticalis, when remnants of same remain, is made possible by simply withdrawing and re-inserting the spoon.

The pressure applied by a delicate rubber spoon excludes the fear of injuring the surface of the epithelium of the cornea, as was satisfactorily established by the two hundred and thirty cases above alluded to. The course of the knife in this method is somewhat more oblique. A. Weber's experiments proved that an exact closing of large linear wounds was a consequence, and the direction of the channel of the wound is more apt to correspond to the course of exit of the cataract. The height of the flap is enlarged (from 1-6‴ to 1-3‴.) This modification of the linear extraction has established a great progress in this, that not only the advantages of the flap extraction are secured, but surpassed in every respect. All prominent authorities proclaim the modified linear extraction in its present form as the most complete, to the exclusion of all other modes of operation. Its introduction in modern surgery is considered next to the iridectomy in glaucoma, the grandest achievement of the immortal von Graefe. His death in the spring of 1870, was a great loss both to science and humanity. As a surgeon he is perhaps already replaced. (I mention, for example, Hirschberg, in Berlin, Jacobson, in Koenigsberg, and Knapp, in New York.) In vain will we seek his equal in the field of natural history. Men who, as he, in the midst of exhausting activity could still follow grand ideas, return to us only after centuries of time.

